# Supplementary material for: Impact of early life antibiotic and probiotic treatment on gut microbiome and resistome of very-low-birth-weight preterm infants
Source: Nat Commun. 2025 Aug 14;16:7569. doi: 10.1038/s41467-025-62584-2 (PMC12354744; doi:10.1038/s41467-025-62584-2)
Supplement: Supplementary file 1 — Supplementary Information [file 41467_2025_62584_MOESM1_ESM.pdf]

Supplementary Information

Impact of early life antibiotic and probiotic treatment on gut microbiome and resistome of very-low-birth-weight preterm infants

Raymond Kiu[1,2,3*], Elizabeth M. Darby[1,2#], Cristina Alcon-Giner[3#], Antia Acuna-Gonzalez[3#], Anny Camargo[3,4,5], Lisa E. Lamberte[2], Sarah Phillips[3], Kathleen Sim[6], Alexander G. Shaw[6], Paul Clarke[7,8], Willem van Schaik[1,2], J Simon Kroll[6], Lindsay J. Hall[1,2,3,8*]

[1] Department of Microbes, Infection and Microbiomes, School of Infection, Inflammation and Immunology, College of Medicine and Health, University of Birmingham, Birmingham, UK. [2] Institute of Microbiology and Infection, University of Birmingham, Birmingham, UK. [3] Food, Microbiome and Health, Quadram Institute Bioscience, Norwich Research Park, Norwich, UK. [4] Centro de Investigaciones en Microbiología y Biotecnología-UR (CIMBIUR), Facultad de Ciencias Naturales, Universidad del Rosario, Bogotá, Colombia. [5] Health Sciences Faculty, Universidad de Boyacá, Tunja, Colombia. [6] Faculty of Medicine, Imperial College London, London, UK. [7] Norfolk and Norwich University Hospital, Norwich, UK. [8] Norwich Medical School, University of East Anglia, Norwich, UK. [#] These authors have contributed equally to this work. * Corresponding authors.

Supplementary Tables

Supplementary Table 1. Summary of cohort characteristics: NPS cohort vs PS cohort.

**Characteristic**	**NPS (n=19)**	**PS (n=15)**
**Demographics**		
Male	6 (32)	10 (67)
Female	13 (68)	5 (33)
Median birth weight (IQR), g	980 (1000-1325)	1382 (1243-1451)
Median gestation at birth (IQR), weeks + days	28+6 (28+2 - 30+1)	30+0 (29+5 – 30+1)
Mode of delivery		
Caesarean section	17 (79)	7 (47)
Normal vaginal delivery	4 (21)	8 (53)
**Antibiotic use in the first 3 weeks of life**		
Median No. of days of treatment in treated infants (IQR)	3.0 (3.0-5.0)	3.0 (3.0-4.0)
No. of infants without treatment	10 (53)	3 (20)
No. of infants on short-course (1-3 day) treatment	6 (32)	8 (53)
No. of infants on long-course (>3 days) treatment	3 (16)	4 (27)
**Diet**		
Breastmilk/donor breastmilk (week 1)	19 (100)	15 (100)
Breastmilk/donor breastmilk (week 2)	19 (100)	15 (100)
Breastmilk/donor breastmilk (week 3)	18 (95)	12 (80)
**Sample collection**		
Median No. of samples collected (per infant)	3.0	3.0
Week 1 median time point, day (IQR), No. of infants (%)	7.0 (7.0-7.8), 18 (95)	6.0 (5.0-7.0), 11 (73)
Week 2 median time point, day (IQR), No. of infants (%)	14.0 (14.0-14.0), 19 (100)	13.0 (11.5-14.5), 15 (100.0)
Week 3 median time point, day (IQR), No. of infants (%)	21.0 (20.8-21.3), 16 (84)	20.0 (20.0-22.0), 13 (87)
**Sequencing depth**		
Mean No. of sequencing reads	10,578,191	10,869,232
Mean metagenome size (bp)	1,322,088,528	1,358,155,375

Data are presented as No. (%) unless otherwise specified.

Supplementary Figures

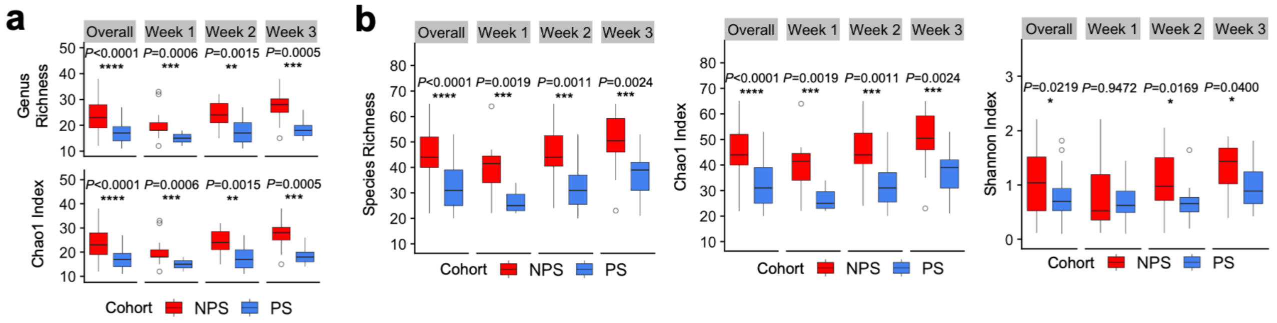

Supplementary Fig. 1 | a, Genus-level gut microbiome richness indices, including genus richness (top) and Chao1 index (bottom), in 34 infants from two cohorts (NPS vs PS), stratified by week. Estimates from both indices were statistically identical. Statistical significance was assessed using a two-sided Wilcoxon test (NPS vs PS stratified by week), with Ben amini–Hochberg correction. **P*<0.05, ***P*<0.01, ****P*<0.001, *****P*<0.0001. **b,** Species-level gut microbiome diversity indices in 34 infants from two cohorts (NPS vs PS), stratified by week, including species richness (absolute species count; left), Chao1 index (species estimation; middle), and Shannon diversity index (right). Statistical significance was assessed using a two-sided Wilcoxon test (NPS vs PS stratified by week), with Benjamini–Hochberg correction. Species richness and Chao1 index yielded identical statistical outcomes.. **P*<0.05, ***P*<0.01, ****P*<0.001, *****P*<0.0001. In **a** and **b**, the box plots represent median (line inside the box), interquartile range (IQR; middle 50% of the data, box height), data within 1.5×IQR (whiskers), and outliers (points).

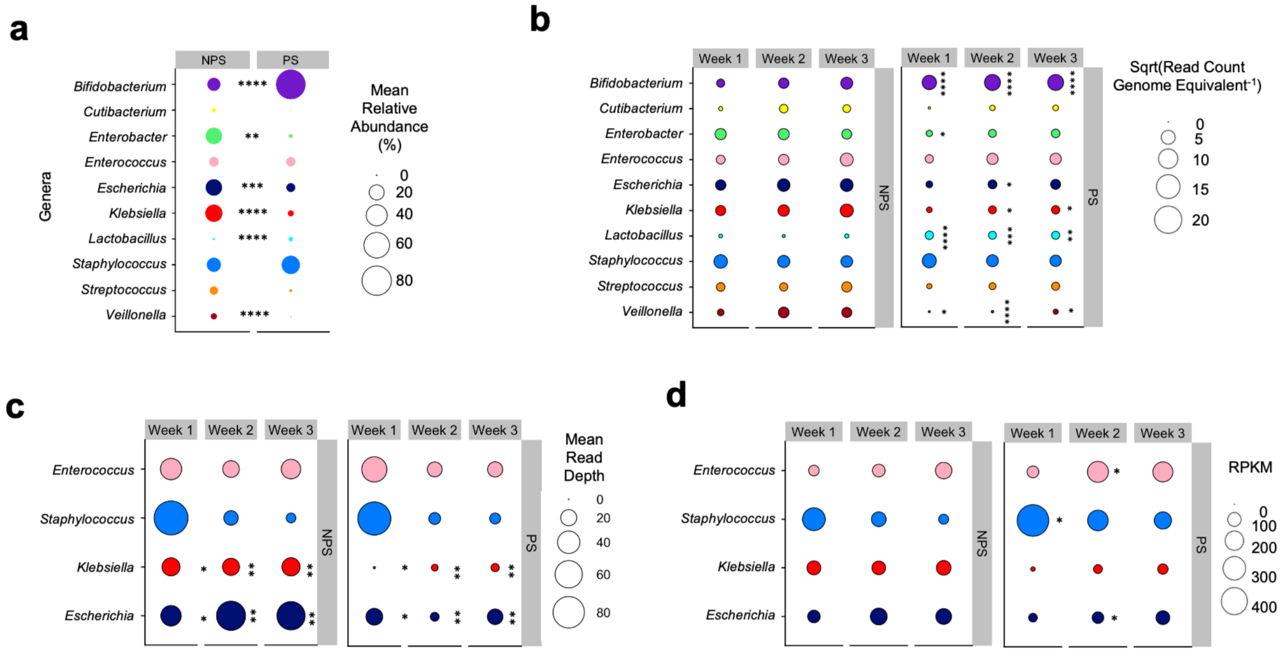

Supplementary Fig. 2 | a, Overall mean relative abundance comparison of the top 10 most abundant bacterial genera in preterm infants (*n*=19 NPS, *n*=15 PS) during the first 3 weeks of life. Statistical significance was assessed using two-sided Wilcoxon test with Benjamini-Hochberg correction. ***P*<0.01, ****P*<0.001, *****P*<0.0001. **b,** Square root of read count per genome equivalent (normalized by genome number in each metagenome) for the top 10 most abundant bacterial genera in both cohorts, shown as bubble plots (stratified by weeks). Significance was assessed using a two-sided Wilcoxon test with Benjamini–Hochberg correction, comparing NPS (*n*=19) vs PS (*n*=15) across weeks. Statistical outcomes were consistent with the mean relative abundance results shown in **Fig. 1h**. **P*<0.05, ***P*<0.01, ****P*<0.001, *****P*<0.0001. **c,** Mean read depth (coverage) comparison of the top four most resistant bacterial genera in both cohorts (*n*=19 NPS, *n*=15 PS). A consistent reduction in *Escherichia* and *Klebsiella* supports the findings shown in **Fig. 1h**. Significance was assessed using a two-sided Wilcoxon test with Benjamini–Hochberg correction, comparing NPS vs. PS across weeks.**P*<0.05, ***P*<0.01. **d,** Reads Per Kilobase per Million mapped reads (RPKM) comparison of the top four most resistant bacterial genera in both cohorts (*n*=19 NPS, *n*=15 PS). Significance was assessed using a two-sided Wilcoxon test with Benjamini–Hochberg correction, comparing NPS vs. PS across weeks. **P*<0.05.

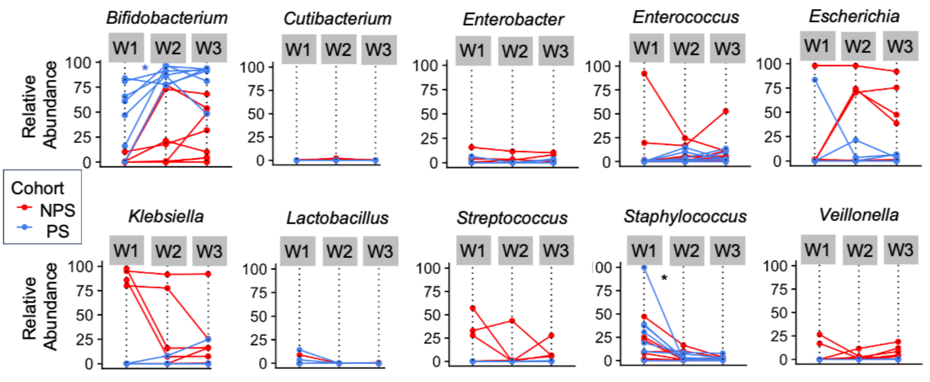

Supplementary Fig. 3 | Intra-cohort paired samples (*n*=9 NPS, *n*=7 PS) from antibiotic-exposed preterm infants were analysed longitudinally over the first three weeks of life to assess the relative abundance of the top 10 bacterial genera. Statistical analysis was performed using the Wilcoxon test with Benjamini–Hochberg correction. *P<0.05. Black asterisks indicate comparisons across all time points, while coloured asterisks denote cohort-specific comparisons.

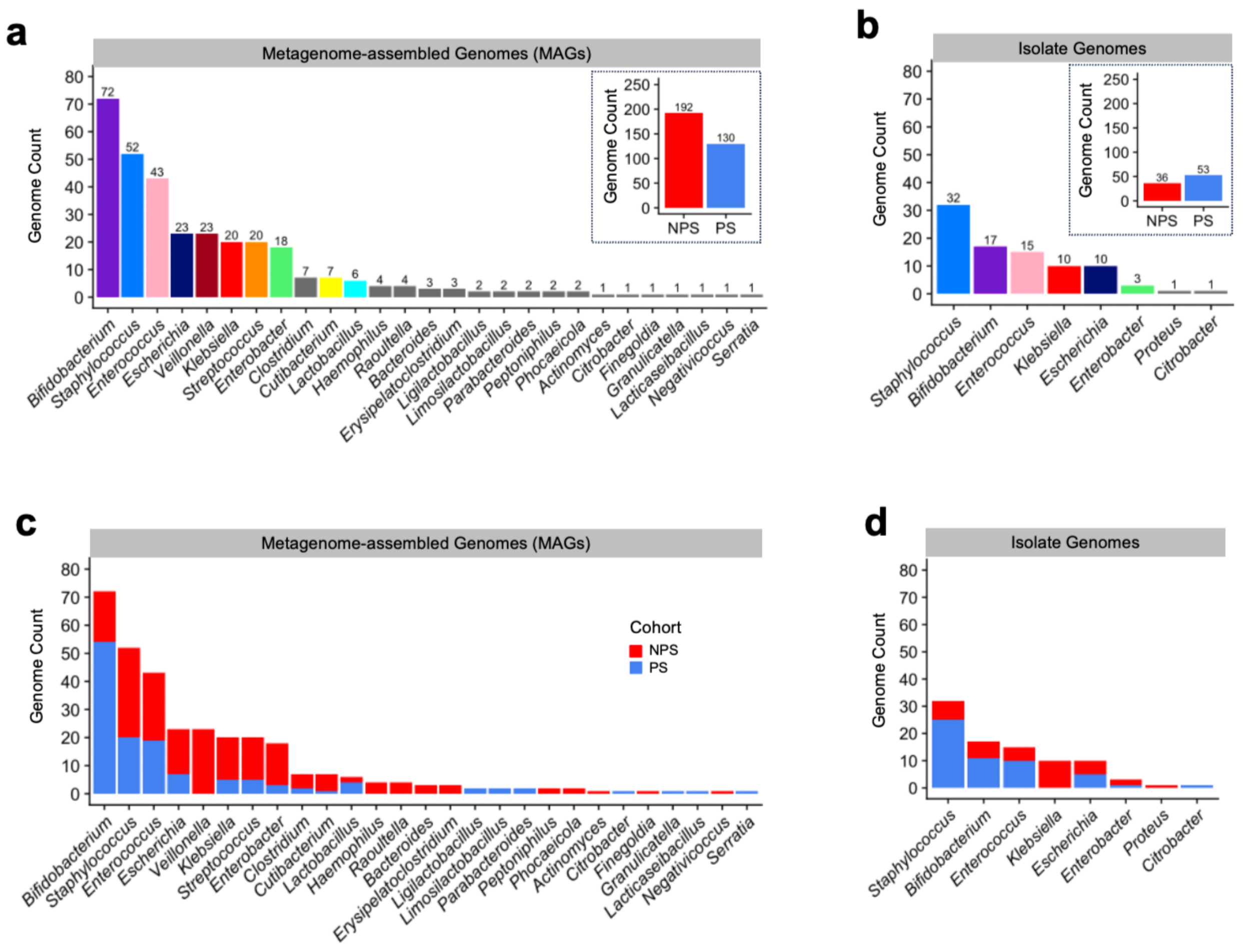

Supplementary Fig. 4 | a, Genome counts of metagenome-assembled genomes (MAGs) from 27 bacterial genera. The inset bar chart indicates the number of MAGs identified in each cohort. Numbers in both charts (on top of each bar) denote genome counts. **b**, Genome counts of pure isolate genomes from 8 bacterial genera. The inset bar chart indicates the number of isolate genomes in each cohort. Small numbers in both charts denote genome counts. **c**, Genome counts of MAGs classified by cohort, with cohort colour-coded. **d**, Genome counts of pure isolate genomes classified by cohort, with cohort colour-coded.

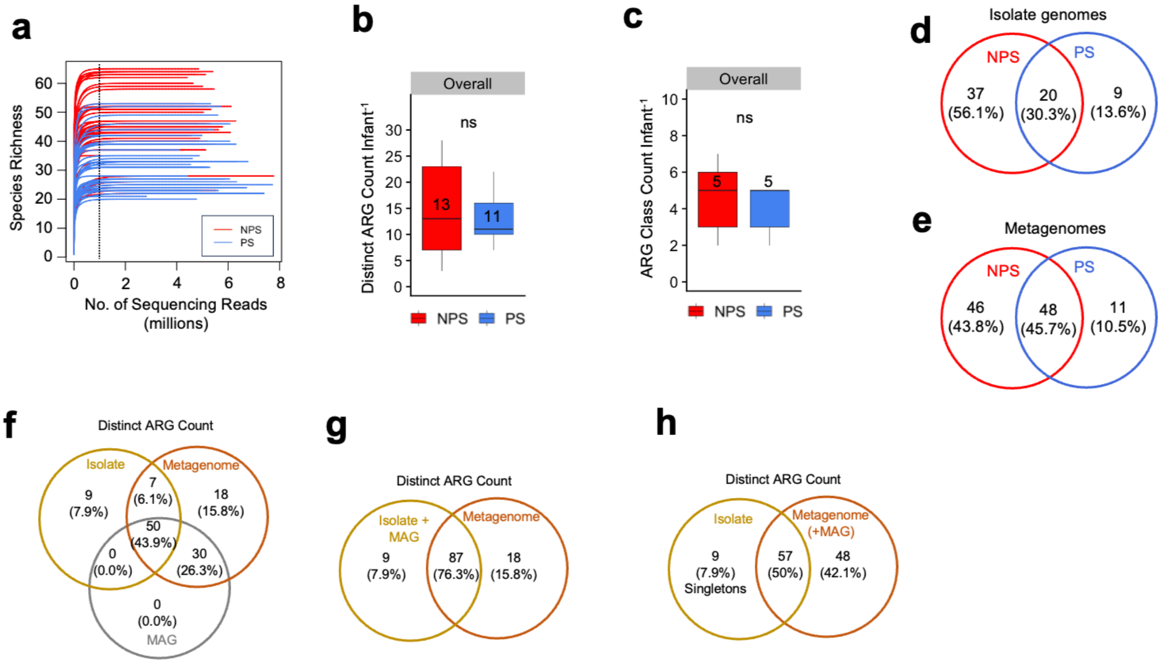

Supplementary Fig. 5 | a, Rarefaction curves constructed from preterm infant metagenome reads (*n*=92), based on species-level taxonomic reads, colour-coded by cohort (*n*=53 NPS vs *n*=39 PS). The dashed line indicates the 1 million read threshold, beyond which all curves appear to plateau, suggesting sufficient sequencing coverage to capture species diversity. **b**, Comparison of the average number of antibiotic resistance genes (ARGs) per infant between NPS and PS cohorts, based on 89 isolate genomes (5 infants per cohort). Numbers within box plots indicate median values. Statistical analysis was performed using a two-sided Wilcoxon test with Benjamini–Hochberg correction. *ns*, not significant. **c**, Comparison of the average number of ARG classes (i.e., antibiotic classes) per infant between NPS (*n*=5) and PS (*n*=5) cohorts, based on 89 isolate genomes. Median values are indicated within the box plots. Statistical analysis was performed using a two-sided Wilcoxon test with Benjamini–Hochberg correction. *ns*, not significant. **d**, Venn diagram comparing ARG counts from isolate genomes (*n*=89) between NPS and PS cohorts. **e**, Venn diagram comparing ARG counts from infant metagenomes (*n*=92) between NPS and PS cohorts (*n*=53 NPS vs *n*=39 PS). **f**, Comparison of shared and unique ARGs from three sources: isolate genomes (Isolate), infant metagenomes (Metagenome), and metagenome-assembled genomes (MAGs). Notably, 9 singleton ARGs were detected exclusively in isolate genomes and not in metagenomic data, underscoring the complementary value of culturomics alongside metagenomic sequencing. **g**, Analysis of shared and unique ARGs between isolate genomes (*n*=89) combined with MAGs (Isolate + MAG) versus infant metagenomes (Metagenome; *n*=92). **h**, Analysis of shared and unique ARGs between isolate genomes (Isolate; *n*=89) versus infant metagenomes (*n*=92) combined with MAGs (Metagenome + MAG). In **b** and **c**, the box plots represent median (line inside the box), interquartile range (IQR; middle 50% of the data, box height), data within 1.5×IQR (whiskers), and outliers (points).

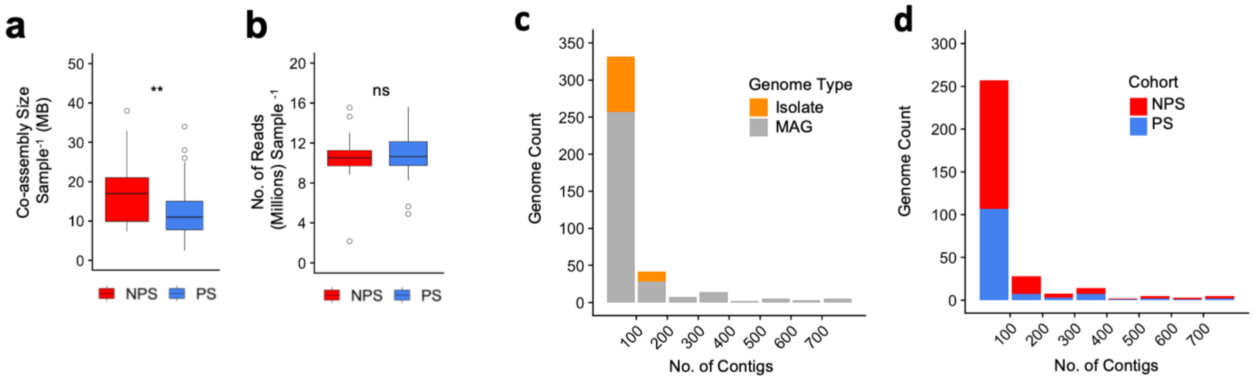

Supplementary Fig. 6 | a, Comparison of average co-assembly size (metagenomic assemblies) between NPS and PS cohorts (*n*=53 NPS vs *n*=39 PS). Statistical significance was assessed using a two-sided Wilcoxon test with Benjamini–Hochberg correction. ***P*<0.01. **b,** Comparison of average sequencing read counts between NPS (*n*=53) and PS (*n*=39) cohorts. Statistical significance was assessed using a two-sided Wilcoxon test with Benjamini–Hochberg correction. *ns,* not significant. **c,** Genome fragmentation assessment based on the number of contigs across all genome types, including metagenome-assembled genomes (MAGs; *n*=322) and pure isolate genomes (*n*=89), colour-coded by genome type. The majority of genomes exhibited high integrity, with fewer than 200 contigs. **d,** Comparison of the number of contigs in MAGs (*n*=322) between NPS and PS cohorts, colour-coded by cohort. In **a** and **b**, the box plots represent median (line inside the box), interquartile range (IQR; middle 50% of the data, box height), data within 1.5×IQR (whiskers), and outliers (points).
