# Supplementary material for: Impact of early life antibiotic and probiotic treatment on gut microbiome and resistome of very-low-birth-weight preterm infants
Source: Nat Commun. 2025 Aug 14;16:7569. doi: 10.1038/s41467-025-62584-2 (PMC12354744; doi:10.1038/s41467-025-62584-2)
Supplement: Supplementary file 2 — Description of Additional Supplementary Files [file 41467_2025_62584_MOESM2_ESM.pdf]

Description of Supplementary Data Files

Supplementary Data 1: Genome statistics and quality information of pure isolate genomes and metagenome-assembled genomes generated in this study.

Supplementary Data 2: Clinical metadata related to the study participants including cohort, antibiotic treatment, delivery mode, gender and gestational age.

Supplementary Data 3: Metadata and statistics related to metagenomic samples used in this study including clinical metadata of participants and sequencing quality.

Supplementary Data 4: Pathway abundance of metagenomic samples stratified by bacterial species.

Supplementary Data 5: Integrated pathway abundance by each metagenomic sample.
