# Supplementary material for: Impact of early life antibiotic and probiotic treatment on gut microbiome and resistome of very-low-birth-weight preterm infants
Source: Nat Commun. 2025 Aug 14;16:7569. doi: 10.1038/s41467-025-62584-2 (PMC12354744; doi:10.1038/s41467-025-62584-2)
Supplement: Supplementary file 8 — Reporting Summary [file 41467_2025_62584_MOESM8_ESM.pdf]

nature portfolio

Corresponding author(s): Prof. Lindsay J Hall
Dr. Raymond Kiu

Last updated by author(s): 15 July 2025

Reporting Summary

Nature Portfolio wishes to improve the reproducibility of the work that we publish. This form provides structure for consistency and transparency in reporting. For further information on Nature Portfolio policies, see our Editorial Policies and the Editorial Policy Checklist.

Statistics

For all statistical analyses, confirm that the following items are present in the figure legend, table legend, main text, or Methods section.

n/a	Confirmed	
☐	☒	The exact sample size (*n*) for each experimental group/condition, given as a discrete number and unit of measurement
☒	☐	A statement on whether measurements were taken from distinct samples or whether the same sample was measured repeatedly
☐	☒	The statistical test(s) used AND whether they are one- or two-sided<br>*Only common tests should be described solely by name; describe more complex techniques in the Methods section.*
☒	☐	A description of all covariates tested
☐	☒	A description of any assumptions or corrections, such as tests of normality and adjustment for multiple comparisons
☐	☒	A full description of the statistical parameters including central tendency (e.g. means) or other basic estimates (e.g. regression coefficient) AND variation (e.g. standard deviation) or associated estimates of uncertainty (e.g. confidence intervals)
☐	☒	For null hypothesis testing, the test statistic (e.g. *F*, *t*, *r*) with confidence intervals, effect sizes, degrees of freedom and *P* value noted<br>*Give P values as exact values whenever suitable.*
☒	☐	For Bayesian analysis, information on the choice of priors and Markov chain Monte Carlo settings
☒	☐	For hierarchical and complex designs, identification of the appropriate level for tests and full reporting of outcomes
☒	☐	Estimates of effect sizes (e.g. Cohen's *d*, Pearson's *r*), indicating how they were calculated

Our web collection on statistics for biologists contains articles on many of the points above.

Software and code

Policy information about availability of computer code

Data collection	No public dataset used in this study. Shotgun metagenome sequencing data newly generated in this study was from stool samples (n=92) of a sub-set of a large preterm infant observational study (n=234) published by the group previously at https://doi.org/10.1016/j.xcrm.2020.100077. Metagenome-assembled genomes (n=322) were computationally constructed using shotgun metagenome data from stool samples (n=92). Pure isolate genomes (n=89) were obtained from preterm infant stool samples via targeted culturomics and whole genome sequencing from infants (n=10). Plasmid-free lab strain Enterococcus faecium 64/3 was previously described in https://doi.org/10.1128/genomea.01275-15 and routinely used in the lab for various plasmid-related experiments. Both Enterococcus faecium ARMA59 and ARMA73 were isolated from preterm infant samples and whole-genome sequenced in this study, as well as other transformants.
Data analysis	1) fastp v0.20.0 2) Unicycler v0.4.9b 3) SPAdes v3.11.1 4) Bowtie2 v2.3.4.1 5) SAMtools v1.7 6) Pilon v1.22 7) CheckM v1.1.3 8) gtdb-tk v1.5.1 9) sequence-stats v1.0 10) Prokka v1.14.6 11) KneadData v0.10.0 12) MEGAHIT v1.2.9 13) MetaWRAP v1.3.2 14) MetaBAT v2.12.1 15) MaxBin v2.2.6 16) CONCOCT v1.1.0 17) dRep v3.2.2 18) Kraken v2.1.2 19) Bracken v2.6.2 20) Mashtree v1.2.0 21) iTOL v6 22) Humann v3.0.0 23) Metaphlan v3.0.13 24) ABRicate v1.0.1 25) METABOLIC v4.0 26) MLST v2.19.0 27) Kleborate v2.3.2 28) Waafle v1.0 29) Graphpad Prism v10 30) filtlong v2.9 31) Flye v2.9 32) Unicycler v0.5.0 33) fastANI v1.34 34) R v4.1.2 35) R tidyverse v1.3.1 36) R ggplot2 v3.3.5 37) R ggpubr v0.6.0 38) R Vegan v2.6.2 39) R genoplotr v0.8.11 40) GenoFig v1.1.1 41) R stats v4.1.2 42) LEfSe 43) R dplyr v1.0.2 44) R rstatix v0.6.0 45) iRep v1.1.7 46) Bowtie v2.3.4.1 47) Microbe Census v1.1.0 48) CoverM v0.7.0 49) minimap2 v2.26 50) Medaka v1.11.3. R scripts and source data files associated with data visualisation and statistical analysis are available and shared via GitHub (https://github.com/raymondkiu/Infant-Resistome-Study)

For manuscripts utilizing custom algorithms or software that are central to the research but not yet described in published literature, software must be made available to editors and reviewers. We strongly encourage code deposition in a community repository (e.g. GitHub). See the Nature Portfolio guidelines for submitting code & software for further information.

Data

Policy information about availability of data

All manuscripts must include a data availability statement. This statement should provide the following information, where applicable:

- Accession codes, unique identifiers, or web links for publicly available datasets
- A description of any restrictions on data availability
- For clinical datasets or third party data, please ensure that the statement adheres to our policy

Infant faecal sample metagenome sequencing raw reads (n=92) are publicly available in the NCBI Sequence Read Archive (SRA) under accession no. PRJNA1191223. Sequencing raw reads and draft genome assemblies for 89 pure bacterial isolates generated in the present study are made available in the NCBI SRA and Genome respectively, under accession no. PRJNA119225. Sequencing raw reads and draft genome assemblies from long-read WGS on Enterococcus plasmid transfer study are publicly available in NCBI SRA (for raw sequencing reads) and NCBI Genome (genome assemblies) both under accession no. PRJNA119226. All high-quality metagenome-assembled genomes (n=322) are available and shared via GitHub repository: https://github.com/raymondkiu/Infant-Resistome-Study/tree/main/Metagenome-assembled-genomes

Research involving human participants, their data, or biological material

Policy information about studies with human participants or human data. See also policy information about sex, gender (identity/presentation), and sexual orientation and race, ethnicity and racism.

Reporting on sex and gender	n/a in this study
Reporting on race, ethnicity, or other socially relevant groupings	n/a in this study
Population characteristics	All subjects recruited in this study were premature infants (generally 'healthy') born at gestational age <34 weeks, and resident in the same NICU for study duration. We selected a sub-set (n=34) from the previous large preterm infant observational study (n=234), all exclusively fed with human milk, one cohort received probiotic supplementation, while the other did not. Within each cohort, some infants received antibiotics (benzylpenicillin and/or gentamicin), while others served as non-antibiotic treated controls.
Recruitment	Preterm infants were recruited from 3 different NICUs across England, UK (between 2011-2017) under Norfolk and Norwich University Hospitals NHS Foundation Trust and Imperial Healthcare NHS Trust. Feacal slurry for ex vivo study was prepared from stool samples of 5 infants in PEARL study under Norfolk and Norwich University Hospital NHS foundation.
Ethics oversight	Faecal sample collection from Norfolk and Norwich University Hospital (BAMBI study) was approved by the Faculty of Medical and Health Sciences Ethics Committee at the University of East Anglia (UEA), and followed protocols laid out by the UEA Biorepository (License no: 11208). Faecal sample collection Imperial Healthcare NICUs (NeoM study) was approved by West London Research Ethics Committee (REC) under the REC approval reference number 10/H0711/39. In all cases, medical doctors and nurses recruited infants after parents gave written consent. The PEARL study has been reviewed and agreed by the Human Research Governance Committee at the Quadram Institute Bioscience and the London-Dulwich Research Ethics Committee (reference 18/LO/1703) and received written ethical approval by the Human Research Authority. IRAS project ID number 241880.

Note that full information on the approval of the study protocol must also be provided in the manuscript.

Field-specific reporting

Please select the one below that is the best fit for your research. If you are not sure, read the appropriate sections before making your selection.

☒ Life sciences ☐ Behavioural & social sciences ☐ Ecological, evolutionary & environmental sciences

For a reference copy of the document with all sections, see nature.com/documents/nr-reporting-summary-flat.pdf

Life sciences study design

All studies must disclose on these points even when the disclosure is negative.

Sample size	A sub-set of 92 stool samples from 34 preterm infants underwent shotgun-metagenome sequencing, and 322 metagenome-assembled genomes were generated using these data. A total of 89 pure isolate genomes were obtained via manual bacterial isolation in the wet lab and whole genome sequencing/genome assembly. 2 Enterococcus faecium strains that encoded gentamicin resistance genes were selected as donors for plasmid transfer study as a proof-of-concept study while plasmid-free lab strain 64/3 was used as recipient strain. Feacal slurry for ex vivo study was prepared from stool samples of 5 infants, was whole-genome sequenced for taxonomic profiling.
Data exclusions	1 pure bacterial isolate genome was excluded due to its low genome quality.
Replication	In plasmid transfer study, each co-culturing combination was performed in triplicates (n=3). Experiment was performed once as the aim is to prove the possibility of gentamicin-resistant plasmid among Enterococcus strains using Whole Genome Sequencing approach. No experimental data was excluded.

Randomization	This is not relevant to the study as bacterial strains were allocated to groups for a specific purpose of generating transformants (also some as positive/negative controls) as in the Enterococcus plasmid transfer study.
Blinding	Blinding was not relevant in this study as no subjective analysis performed.

Reporting for specific materials, systems and methods

We require information from authors about some types of materials, experimental systems and methods used in many studies. Here, indicate whether each material, system or method listed is relevant to your study. If you are not sure if a list item applies to your research, read the appropriate section before selecting a response.

Materials & experimental systems

n/a	Involved in the study
☒	☐ Antibodies
☒	☐ Eukaryotic cell lines
☒	☐ Palaeontology and archaeology
☒	☐ Animals and other organisms
☐	☒ Clinical data
☒	☐ Dual use research of concern
☒	☐ Plants

Methods

n/a	Involved in the study
☒	☐ ChIP-seq
☒	☐ Flow cytometry
☒	☐ MRI-based neuroimaging

Clinical data

Policy information about clinical studies

All manuscripts should comply with the ICMJEguidelines for publication of clinical research and a completedCONSORT checklist must be included with all submissions.

Clinical trial registration	n/a
Study protocol	Faecal sample collection from Norfolk and Norwich University Hospital (BAMBI study) was approved by the Faculty of Medical and Health Sciences Ethics Committee at the University of East Anglia (UEA), and followed protocols laid out by the UEA Biorepository (License no: 11208). Faecal sample collection Imperial Healthcare NICUs (NeoM study) was approved by West London Research Ethics Committee (REC) under the REC approval reference number 10/H0711/39. The PEARL study has been reviewed and agreed by the Human Research Governance Committee at the Quadram Institute Bioscience and the London-Dulwich Research Ethics Committee (reference 18/LO/1703) and received written ethical approval by the Human Research Authority. IRAS project ID number 241880. In all cases, medical doctors and nurses recruited infants after parents gave written consent.
Data collection	Medical data and stool samples were collected by medical doctors and research nurses during the study period.
Outcomes	n/a

Plants

Seed stocks	*Report on the source of all seed stocks or other plant material used. If applicable, state the seed stock centre and catalogue number. If plant specimens were collected from the field, describe the collection location, date and sampling procedures.*
Novel plant genotypes	*Describe the methods by which all novel plant genotypes were produced. This includes those generated by transgenic approaches, gene editing, chemical/radiation-based mutagenesis and hybridization. For transgenic lines, describe the transformation method, the number of independent lines analyzed and the generation upon which experiments were performed. For gene-edited lines, describe the editor used, the endogenous sequence targeted for editing, the targeting guide RNA sequence (if applicable) and how the editor was applied.*
Authentication	*Describe any authentication procedures for each seed stock used or novel genotype generated. Describe any experiments used to assess the effect of a mutation and, where applicable, how potential secondary effects (e.g. second site T-DNA insertions, mosiacism, off-target gene editing) were examined.*
